# High-Resolution Characterization of the Bacterial Microbiome in Chili Pepper Powder Using SMRT Sequencing

**DOI:** 10.4014/jmb.2506.06003

**Published:** 2025-10-15

**Authors:** Hae-Won Lee, Ji-Hyoung Ha

**Affiliations:** 1 Department of Food Bioengineering, Jeju National University, Jeju 63243, Republic of Korea; 2 Hygienic Safety ·Materials Research Group, World Institute of Kimchi, Gwangju 61755, Republic of Korea

**Keywords:** Bacterial microbiome, single-molecule real-time sequencing, chili pepper, *Capsicum annuum*

## Abstract

Chili pepper powder is an essential ingredient of Korean cuisine and kimchi products. As such, ensuring its microbiological safety is crucial from a public health perspective. In this study, the bacterial microbiomes of Korean and Chinese chili pepper powder samples were compared using high-resolution single-molecule real-time sequencing. Nine samples each from Korea and China were analyzed, yielding high-quality sequencing data with N50 values exceeding 1,460 bp and quality scores greater than Q30. Based on alpha diversity analysis, Chinese samples exhibited higher microbial diversity, whereas Korean samples had a more selective and clustered microbial structure, composed predominantly of *Bacillus*. Taxonomically, Chinese samples contained a wider range of genera, including *Pantoea*, *Weissella*, and *Klebsiella*, in contrast, Korean samples were dominated by *Firmicutes*, primarily *Bacillus* species. Although *Salmonella enterica* was detected in some Korean samples, viability was not confirmed. This may have indicated residual DNA or viable but nonculturable cells due to the low water activity of chili powders. Beta diversity analysis indicated moderate separation between the two groups, with Korean samples showing tighter clustering and Chinese samples displaying greater variability. Linear discriminant analysis effect size identified *Bacillus altitudinis* and *Bacillus safensis* as biomarkers for Korean samples, and *Pantoea agglomerans* and *Weizmannia coagulans* as biomarkers for Chinese samples. Overall, despite slightly lower microbial diversity, Korean chili pepper powder samples exhibited a more stable microbial community structure. Selection of chili pepper powder characterized by a stable and controlled microbiome could play an essential role in promoting the hygienic production and microbial safety of kimchi.

## Introduction

Chili pepper (*Capsicum annuum*) is an essential ingredient of Korean cuisine [1], playing a crucial role in traditional Korean dishes, particularly kimchi [2]. Chili pepper is mostly used in its powdered form in kimchi, and it contributes to the distinctive spiciness, vibrant red color, and depth of flavor, characteristic of this fermented dish. Beyond its culinary applications, chili pepper is valued for its bioactive compounds, including capsaicinoids, flavonoids, and carotenoids, which offer various health benefits such as antioxidant, anti-inflammatory, and antimicrobial properties [3]. Considering its widespread use, ensuring microbiological safety of chili pepper powder is critical, as contamination may occur during agricultural production and post-harvest handling, with improper drying or storage further promoting microbial proliferation [4-6]. The presence of several bacterial species, including foodborne pathogens and spoilage organisms, has been reported in chili pepper powder [6-8].

Kimchi fermentation is a dynamic process influenced by various factors, including the microbiota associated with its ingredients. Chili pepper powder can significantly affect the fermentation, influencing the growth of lactic acid bacteria and other beneficial microbes essential for the ripening and preservation of kimchi [9, 10]. Microbial contaminants in chili pepper may introduce undesired pathogens into the fermentation system, leading to potential safety issues [11-13]. For example, evidence from studies on fermented chili indicates that deterioration or compromise of raw materials reshapes microbial profiles and degrades product quality [12]. Therefore, microbiological contamination carried by chili pepper powder could perturb early lactic acid bacteria succession, acidification dynamics, and flavor development during kimchi fermentation, underscoring the need to monitor the ingredient microbiomes.

In this study, we aimed to investigate the bacterial microbiomes of chili pepper powder samples commonly used in kimchi production, with a particular focus on comparing microbial communities between powders sourced from Korea and China, *i.e.*, the two major suppliers of chili pepper used in Korean kimchi manufacturing. Specifically, this study explored whether chili pepper powders from different origins were associated with a tradeoff between microbiome stability (characterized by community homogeneity and dominance of beneficial taxa) and diversity (which may enhance functional potential but also increase the risk of opportunistic or pathogenic microorganisms occurring). By investigating this stability‒diversity balance, we aimed to provide insights that can inform microbial risk management and quality control strategies in kimchi production.

## Materials and Methods

### Sample Preparation

Korean and Chinese chili pepper powders were purchased from a kimchi manufacturer in Korea in August 2022. Nine samples each of Korean and Chinese chili pepper powders were collected separately. The samples were stored at room temperature in a dark place until further processing. To preserve DNA integrity before extraction, a DNA/RNA shield (Zymo Research, USA) was added to the samples.

### DNA Extraction

DNA was extracted from the samples using the DNeasy PowerSoil Kit (Qiagen, Germany). The extracted DNA was quantified using the Quant-IT PicoGreen Assay Kit (Invitrogen, UK), following the manufacturer’s instructions, and used for analyzing the bacterial microbiome in chili pepper powder samples.

### Amplicon-Based Metagenomic Sequencing of Bacterial Microbiome

For analyzing the bacterial microbiome in chili pepper powder samples, bacterial libraries were prepared via polymerase chain reaction (PCR) amplification using with a primer set (27F, 5'-AGRGTTYGATYMTGGCTCAG- 3'; 1492R, 5'-GGTTACCTTGTTACGACTT-3') targeting the full-length 16S rRNA gene. PCR conditions were as follows: initial denaturation at 94°C for 3 min, followed by 25 cycles of denaturation at 95°C for 30 sec, annealing at 55°C for 30 sec, extension at 72°C for 30 sec, and a final extension at 72°C for 5 min. The generated PCR products were purified using AMPure beads (Agencourt Bioscience, USA), and the quantity and size of the purified PCR products were verified using the Quant-IT PicoGreen Assay Kit (Invitrogen). The size distribution of the template was assessed using the Agilent DNA 12000 Kit (Agilent Technologies, USA). Pooled PCR products for library preparation were processed for PacBio Sequel sequencing using the PacBio DNA Template Prep Kit 1.0 (Pacific Biosciences, USA). Single-molecule real-time (SMRT) sequencing libraries were prepared using the PacBio DNA Sequencing Kit 4.0 and eight SMRT cells (Pacific Biosciences).

The SMRT sequencing was performed using a PacBio Sequel II system (Pacific Biosciences) in accordance with the manufacturer’s instructions. In brief, 10-hour movies were captured for each SMRT cell (Pacific Biosciences). Subsequent steps were carried out based on the PacBio Sample Net-Shared Protocol (https://www.pacb.com/). Circular consensus sequencing (CCS) reads, such as raw sequence reads, were processed using the SMRT analysis software (version 2.3, Pacific Biosciences). Short CCS reads and those with zero-quality bases, considered as sequencing errors, were removed.

### Taxonomic and Statistical Analyses

Bacterial metagenomic sequence reads were classified into amplicon sequence variants using the DADA2 pipeline [14] for taxonomic analysis, and annotated with BLAST+ v2.9.0 against the National Center for Biotechnology Information (NCBI) 16S ribosomal RNA database. Assignments were cross-checked against the Greengenes v13.5 16S rRNA reference.

Statistical analysis was performed using MicrobiomeAnalyst 2.0 [15]. Data normalization was performed for the total sum scaling, and the alpha diversity was assessed using the Shannon index employing the Phyloseq package [16]. Abundance profiling as stacked bar charts for phylum, genus and species levels was performed by calculating the relative percentage abundance, and less than 50 taxa were merged. The beta diversity of samples as principal coordinate analysis (PCoA) was used and set to Bray-Curtis dissimilarities and permutational multivariate analysis of variance. Hierarchical clustering was performed using Bray–Curtis distances with Ward’s linkage method. Linear discriminant analysis effect size (LEfSe) analysis based on taxonomic classification was performed with a P-value cutoff of 0.1 and a log linear discriminant analysis (LDA) score threshold of 2.

### Data Availability

The sequencing reads of bacterial microbiomes of chili pepper powder samples were deposited to the NCBI under BioProject ID PRJNA1249255. The metadata for each sample can be accessed using SRR33085185, SRR33085184, SRR33085183, SRR33085182, SRR33085181, SRR33085180, SRR33085194, SRR33085193, SRR33085192, SRR33085191, SRR33085179, SRR33085178, SRR33085195, SRR33085186, SRR33085187, SRR33085188, SRR33085189, and SRR33085190.

## Results and Discussion

### Amplicon-Based Metagenomic Sequencing of Bacterial Microbiomes

SMRT sequencing produced high-quality, full-length 16S rRNA gene reads across all analyzed samples (CCS N50: 1.46–1.51 kb; average read quality Q30–Q31). After trimming, Korean datasets averaged 57.25 Mbp (range: 49.6–65.2 Mbp) and 38,345 reads (range: 31,888–43,513), and Chinese datasets averaged 50.01 Mbp (range: 40.0–60.0 Mbp) and 33,648 reads (range: 26,940–40,370). Quality metrics were comparable between groups (Table S1).

The high-quality sequencing results confirmed the reliability of SMRT sequencing for the analysis of bacterial microbiome in Korean and Chinese samples. The Korean samples showed a slightly higher sequencing yield (57.3 Mbp; 38,345 reads) compared with that for the Chinese samples (50.0 Mbp; 33,648 reads); however, both the groups exhibited high read N50 values (≥1,460 bp) and quality scores (≥Q30), indicating accurate taxonomic classification. These results demonstrated the suitability of SMRT sequencing for analyzing the bacterial microbiome of chili pepper powder samples, effectively capturing complex bacterial communities with high accuracy.

### Alpha Diversity of Bacterial Microbiomes in Korean and Chinese Chili Pepper Powder Samples

**Fig. 1:**
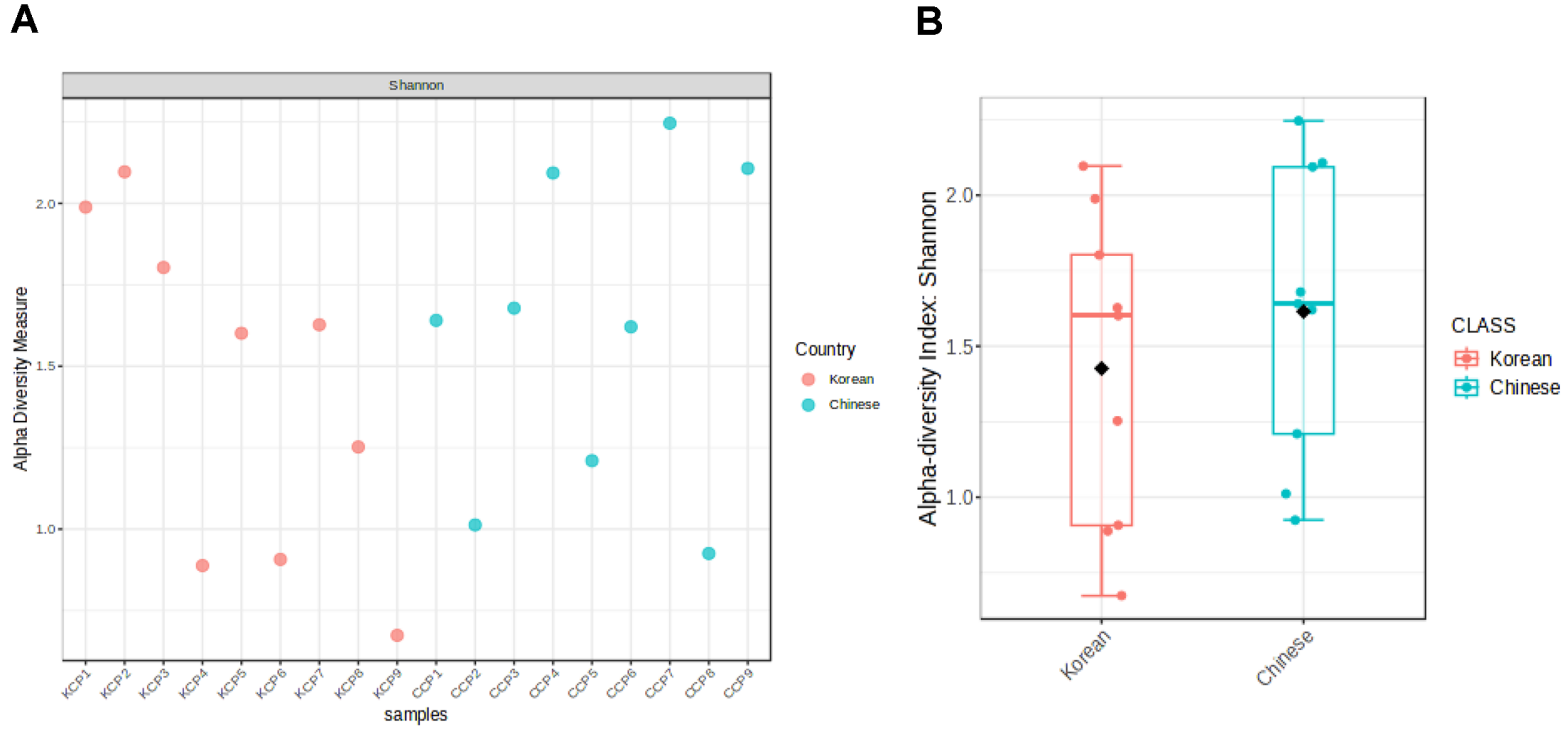
Alpha diversity determined using the Shannon diversity index of bacterial microbiomes in Korean and Chinese chili pepper powder samples. (**A**) Scatter plot showing the Shannon index values for individual samples. (**B**) Box plot comparing the Shannon index distributions between the Korean and Chinese samples. The median, interquartile range, and outliers are represented. KCP, Korean chili pepper powder; CCP, Chinese chili pepper powder.

The alpha diversity of the bacterial microbiome in Korean and Chinese chili pepper powder samples was assessed using the Shannon diversity index, which measures microbial richness and evenness within individual samples. The Shannon index values for each sample are presented in Fig. 1A, and the overall comparison between Korean and Chinese samples is summarized in Fig. 1B. Shannon index analysis revealed that Chinese chili pepper powders generally had higher microbial diversity and a wider range of values, whereas Korean samples exhibited lower diversity with more clustered distributions (Fig. 1B).

The observed differences in alpha diversity determined as the Shannon index were indicative of potential variations in environmental factors, processing methods such as drying techniques, agricultural practices, or storage conditions between Korean and Chinese samples [11, 17, 18]. In addition, variation within each group was observed, indicating inter-sample differences in microbial composition.

### Taxonomic Composition of Bacterial Microbiomes in Korean and Chinese Chili Pepper Powder Samples

**Fig. 2:**
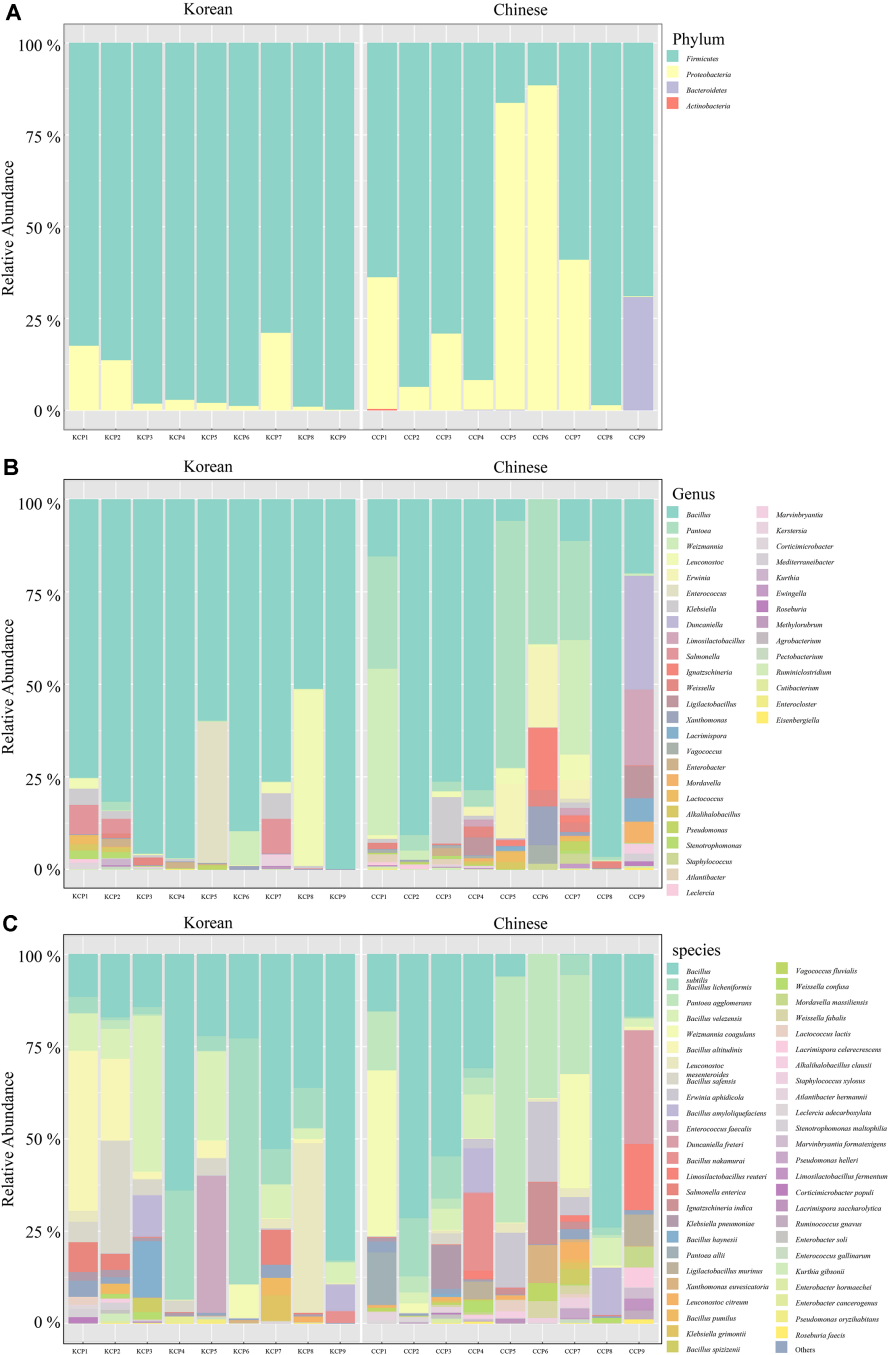
Relative abundance of bacterial taxa in Korean and Chinese chili pepper powder samples. Relative abundance at the phylum (**A**) genus (**B**) and species (**C**) levels. KCP, Korean chili pepper powder; CCP, Chinese chili pepper powder.

The taxonomic composition of bacterial microbiomes in Korean and Chinese chili pepper powders was profiled at the phylum, genus, and species levels (Fig. 2). Long-read SMRT sequencing enabled high-resolution classification and discrimination between sources. At the phylum level (Fig. 2A), both groups were dominated by *Firmicutes* (mean > 70%) followed by *Proteobacteria*. Chinese samples showed higher *Proteobacteria* abundance (≤ 40% in CCP5, CCP6, and CCP7) and, in several cases (CCP4, CCP5, and CCP9), notable Bacteroidetes, which were rarely observed in Korean samples (*i.e.*, only KCP6). At the genus level (Fig. 2B), *Bacillus* dominated both groups, typically exceeding 50% in Korean samples; whereas, Chinese powders contained higher relative abundances of *Pantoea*, *Weissella*, *Enterobacter*, and *Klebsiella*, together approaching 30%. At the species level (Fig. 2C), *Bacillus* was prevalent in samples from both origins, but was more concentrated in Korean samples; Chinese powders included a broader set, such as *Pantoea agglomerans*, *Klebsiella pneumoniae*, and *Enterobacter hormaechei*, *Salmonella enterica* was detected in several Korean samples (KCP1, KCP2, KCP7, and KCP8), but not in Chinese samples.

The observed differences in microbial composition between Korean and Chinese chili pepper powders likely reflected variations in cultivation, drying and milling, and storage practices across supply chains [11, 17, 18]. Prior 16S rRNA gene profiling of Mexican chili powder reported pronounced dominance of *Firmicutes* and, in a subset of samples, *Proteobacteria*; whereas, Actinobacteria and Bacteroidetes were detected at low or sample-restricted levels [6]. This supports the premise that detection of Bacteroidetes in chili powder is not atypical, although its abundance is sample dependent. Additionally, supply-chain factors such as drying, storage, handling, and batch provenance affect microbial contamination in low-water-activity spices [11, 19], which likely contributed to the sample-specific differences observed in the present study. Detection of taxa of concern, most notably *S. enterica* in some of the Korean samples, underscores the need for targeted microbiological monitoring and batch-level quality control in chili pepper powder production and distribution [19]. The absence of *S. enterica* in Chinese samples may have reflected differences in sanitation practices or batch-level variability. Because chili powder is a low-water-activity matrix, DNA-based signals cannot distinguish residual DNA from viable cells. The detected pathogens may have been present as DNA remnants or in viable but nonculturable (VBNC) states, and should therefore be interpreted as potential rather than confirmed hazards [20-25]. Korean powders tended to show more homogeneous, *Bacillus*-dominated communities; whereas, Chinese powders exhibited greater diversity and variability, which could increase opportunities for opportunistic taxa to occur. Future work integrating viability assays, such as culture enrichment, targeted quantitative PCR (qPCR) or droplet digital PCR (ddPCR), and viability-PCR using propidium monoazide (PMA) or ethidium monoazide (EMA), would more accurately link community structure to safety outcomes.

### Beta Diversity of Bacterial Microbiomes of Korean and Chinese Chili Pepper Powder Samples

**Fig. 3:**
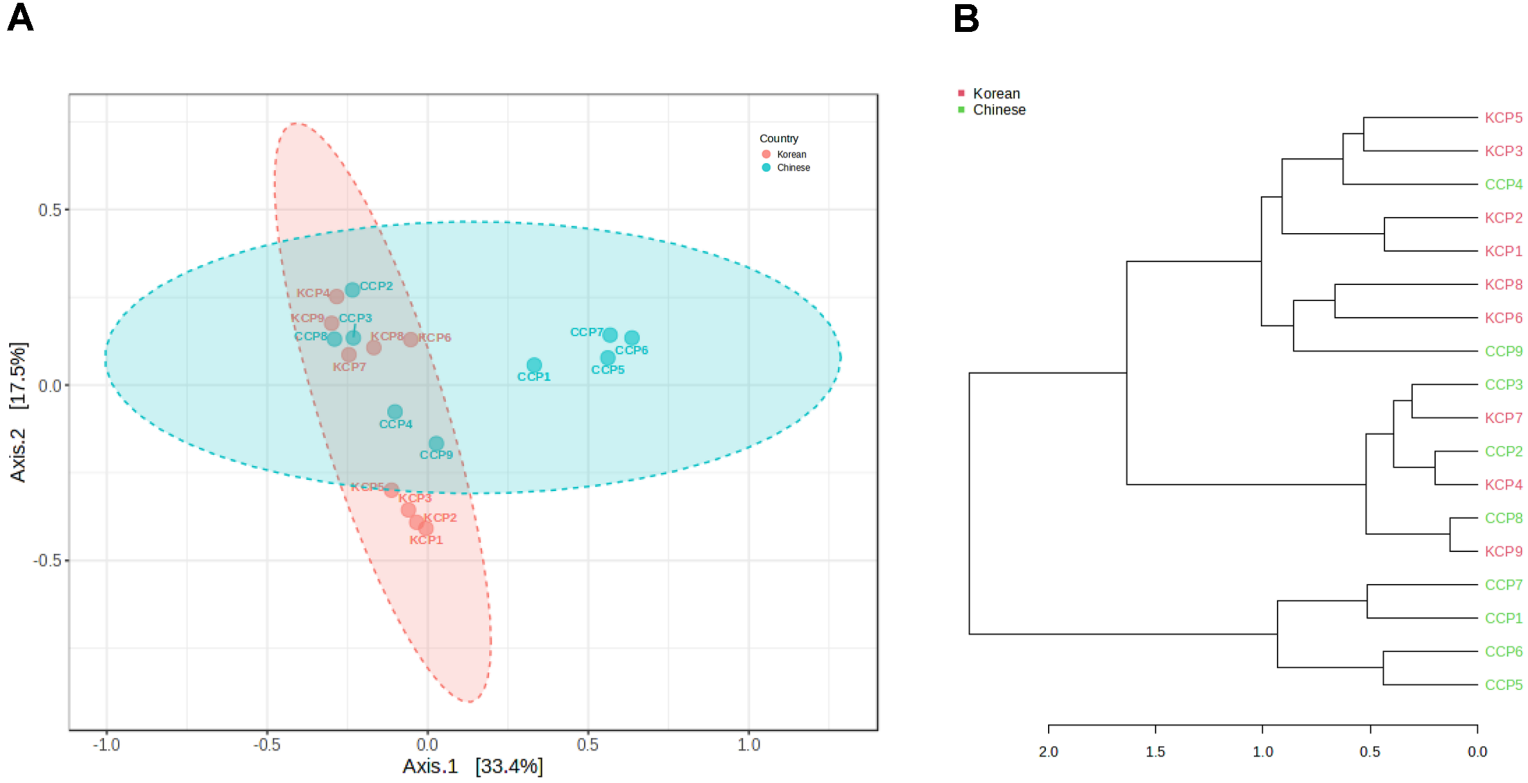
Principal coordinate analysis and hierarchical clustering dendrogram of beta diversity analysis of bacterial microbiomes in Korean and Chinese chili pepper powders. (**A**) Principal Coordinate Analysis (PCoA) plot (pseudo-F = 2.20, R^2^ = 0.121, *p* = 0.039). (**B**) Hierarchical clustering dendrogram. KCP, Korean chili pepper powder; CCP, Chinese chili pepper powder.

**Fig. 4:**
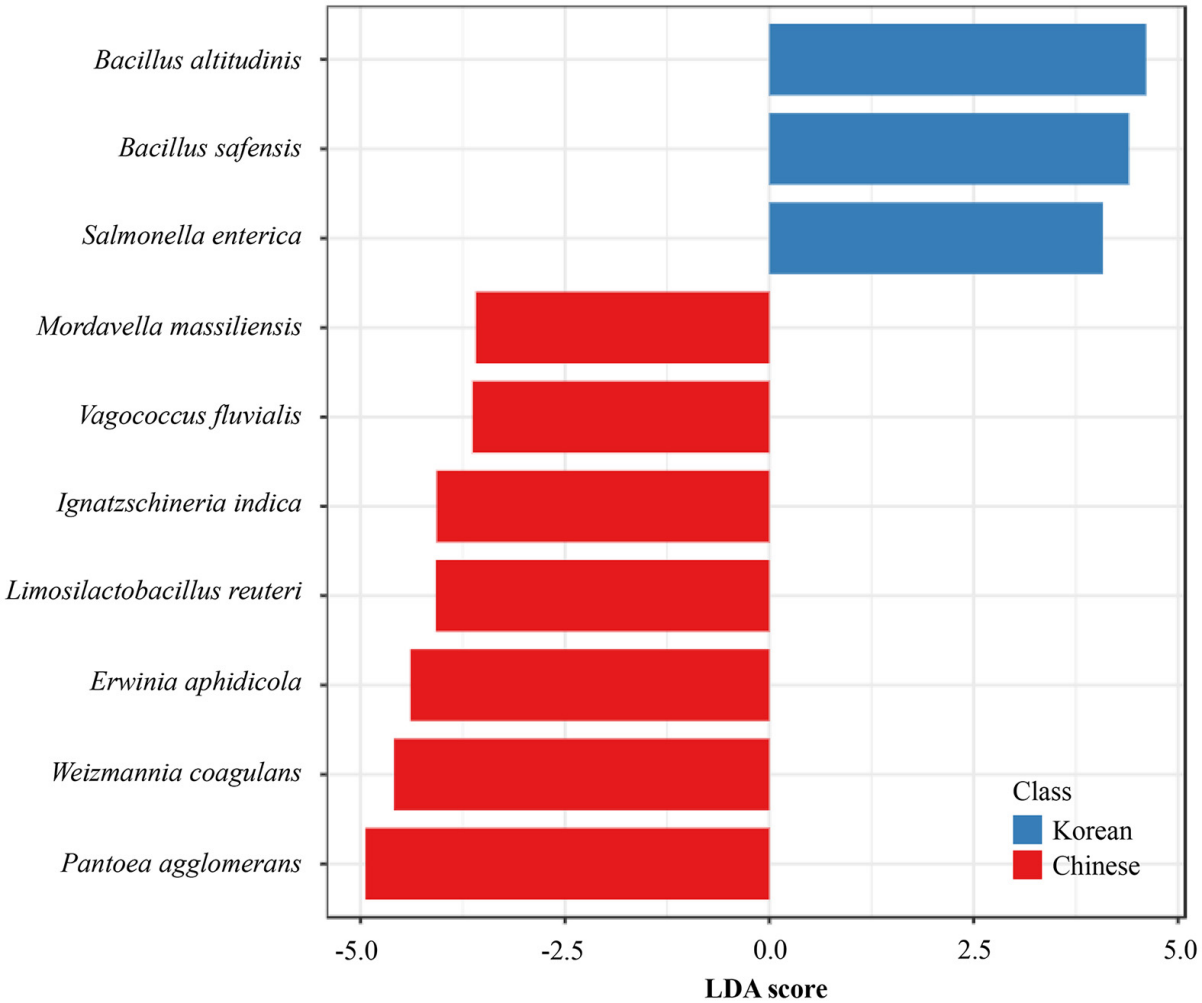
Linear discriminant analysis effect size (LEfSe) analysis identifying differentially abundant bacterial taxa between Korean and Chinese chili pepper powder samples. The top 10 taxa with the highest linear discriminant analysis (LDA) scores are shown. Positive LDA scores indicate bacterial taxa enriched in Korean samples (blue bars), whereas negative LDA scores indicate taxa more abundant in Chinese samples (red bars).

PCoA based on Bray–Curtis dissimilarities showed a reproducible separation between Korean and Chinese chili pepper powder microbiomes (Fig. 3A). The first two axes explained 33.4% (axis 1) and 17.5% (axis 2) of the variance, indicating nontrivial differences in community composition. This separation was further supported by PERMANOVA, which confirmed significant differences between groups (pseudo-F = 2.20, R^2^ = 0.121, *p* = 0.039). Korean samples formed a relatively tight cloud of points, whereas Chinese samples were more widely dispersed, particularly along axis 1. The 95% confidence ellipses reinforced this pattern by showing distinct group centroids with partial overlap at the margins. Hierarchical clustering using Bray–Curtis distances with Ward linkage corroborated the ordination (Fig. 3B). Most samples grouped by origin, with Korean samples forming compact subclusters and Chinese samples showing longer branch lengths and broader cluster breadths. The proximities of several cross groups were observed, for example KCP7 with CCP3, KCP4 with CCP2, and KCP9 with CCP8, consistent with local convergence of community profiles. To connect these patterns to specific taxa, LEfSe was used to identify discriminative biomarkers (Fig. 4). Korean samples were enriched in *Bacillus altitudinis* (LDA 4.61) and *Bacillus safensis* (LDA 4.61), whereas Chinese samples were enriched in *P. agglomerans* (absolute LDA 4.94) and *Weizmannia coagulans* (absolute LDA 4.59). These biomarkers help explain the direction and structure of the ordination and clustering, supporting the view that samples from each origin were characterized by a distinct set of dominant taxa that drove group separation.

The separation was modest yet consistent across both PCoA and hierarchical clustering, indicating environmentally driven microbiome divergence. The concordance between the ordination and the dendrogram indicated that Korean and Chinese chili pepper powders harbored distinct community structures. The compact distribution of Korean samples was consistent with relatively uniform processing and storage, and these samples had a stable community dominated by *Bacillus* lineages. In contrast, the broad spread of Chinese samples suggested heterogeneous inputs and exposures during cultivation, post-harvest handling, drying, storage, and distribution [11, 17, 18]. The LEfSe biomarkers provided biological context. *Pantoea* is frequently associated with plant surfaces and post-harvest environments [26, 27]; *Weizmannia* represents spore-forming bacteria that tolerate heat and desiccation [28], and; both these genera are compatible with greater environmental and process exposure. The small number of cross-group neighbors underscored that factors beyond origin also shaped communities, including harvest timing, drying mode, storage vessel and duration, blending of lots, particle size, and distribution conditions [11, 17, 18]. Because chili powder has low water activity, growth is constrained. As explained earlier, DNA-based detection will nevertheless record signals from residual DNA and from cells in VBNC states; therefore, potential risk should be interpreted cautiously rather than as evidence of active proliferation [20-25]. Practically, combining beta-diversity metrics with biomarker tracking offers a monitoring frame for production. Maintaining a *Bacillus*-centered stability signal while monitoring exposure-linked sentinel taxa such as *Pantoea* and *Weizmannia* can help detect early deviations and trigger corrective action [26-28].

### Comparison of Bacterial Microbiomes between Korean and Chinese Chili Pepper Powder Samples

The comparative analysis of bacterial microbiomes revealed distinct microbial landscapes between the Korean and Chinese chili pepper powder samples. The Korean samples exhibited lower alpha diversity, reflecting a controlled microbial community shaped by different cultivation, processing, storage practices, and distribution. The consistent dominance of *Bacillus* spp., alongside the compact clustering patterns observed in diversity analyses, pointed to a uniform and resilient microbial structure. In contrast, the Chinese samples exhibited higher microbial richness and greater community variability, possibly arising from broader environmental exposure during cultivation, post-harvest handling, and distribution. Taxonomic profiling further highlighted these differences, with *Firmicutes*, particularly *Bacillus* species, prevailing in Korean samples, and *Proteobacteria* and *Bacteroidetes* predominant in Chinese samples. Although the detection of *Salmonella enterica*, *Pantoea agglomerans*, and *Weizmannia coagulans* in some Korean and Chinese samples necessitates caution, its relevance should be interpreted in light of the low-water-activity conditions that suppress bacterial proliferation. Therefore, chili pepper powder characterized by a stable and controlled microbiome could promote hygienic production and microbial safety of kimchi. Accordingly, prioritizing chili pepper powders that exhibit a stable and well-controlled microbiome can support hygienic production and microbial safety of kimchi. Such a profile can function as a baseline for ingredient selection and longitudinal surveillance. Shifts toward higher diversity or increased representation of environmental taxa can be interpreted as early signals of process drift, prompting review and refinement of raw-material handling, drying, storage, and distribution. Framing quality oversight through this stability and diversity perspective also helps align supplier management, monitoring intensity, and corrective actions with the inferred microbiological risk within existing quality control (QC) and hazard analysis and critical control points (HACCP) programs.

## Conclusion

In this study, we compared the bacterial microbiome of Korean and Chinese chili pepper powder samples using high-resolution, long-read SMRT sequencing. Significant differences in bacterial diversity and community structure were observed between the two groups. Although *Firmicutes* was predominant in both Korean and Chinese samples, the Chinese samples exhibited higher relative abundance of *Proteobacteria* and contained diverse genera, such as *Pantoea*, *Weissella*, *Enterobacter*, and *Klebsiella*. Notably, potential foodborne pathogens such as *S. enterica* were detected in chili pepper powder, underscoring the importance of stringent microbiological safety measures during production and storage. Given the low water activity typical of chili pepper powders, the detected bacteria might have represented residual DNA or cells in VBNC states, suggesting potential risks rather than active contamination. Therefore, continuous microbial monitoring, coupled with proper hygienic practices and optimized processing conditions, is crucial to ensuring microbial safety and product quality in chili pepper powder manufacturing. In addition to regular monitoring to maintain stable profiles, follow-up of taxa of concern should be prioritized through enrichment cultures and viability-based molecular assays such as PMA or EMA combined with qPCR or ddPCR. Future research employing functional genomics and metabolic profiling could further elucidate the effect of these microbial communities on food safety, spoilage potential, and fermentation quality, providing deeper insights into microbiological management strategies.

## Supplemental Materials

Supplementary data for this paper are available on-line only at http://jmb.or.kr.
