# Supplementary material for: High-Resolution Characterization of the Bacterial Microbiome in Chili Pepper Powder Using SMRT Sequencing
Source: J Microbiol Biotechnol. 2025 Oct 15;35:e2506003. doi: 10.4014/jmb.2506.06003 (PMC12549223; doi:10.4014/jmb.2506.06003)
Supplement: Supplementary file 1 [file jmb-35-e2506003-supple.pdf]

J. Microbiol. Biotechnol. 2025. 35: e2506003

https://doi.org/10.4014/jmb.2506.06003

Table S1. Sequencing statistics of bacterial microbiomes from Korean and Chinese chili pepper powder samples. The table summarizes sequencing quality metrics, including HiFi read bases, total read counts, read N50 values, average read length, read quality scores (Q-scores), and average pass counts for each sample. All samples passed the DNA and 16S rRNA gene sequencing quality control (QC).

Sample names	Read bases	Read counts	N50 values	Average read lengths	Average read qualities
KCP1	55,926,149	37,539	1,489	1,460	Q31
KCP2	62,811,766	41,791	1,502	1,460	Q30
KCP3	65,155,424	43,513	1,497	1,508	Q31
KCP4	57,988,999	39,089	1,483	1,460	Q30
KCP5	52,832,629	35,484	1,488	1,460	Q31
KCP6	60,182,237	40,404	1,489	1,460	Q30
KCP7	55,374,395	37,308	1,484	1,460	Q31
KCP8	55,409,882	37,158	1,491	1,460	Q30
KCP9	49,612,566	32,823	1,511	1,511	Q30
CCP1	39,965,738	26,940	1,483	1,460	Q31
CCP2	44,365,409	29,913	1,483	1,460	Q31
CCP3	56,292,930	37,974	1,482	1,460	Q31
CCP4	53,593,669	36,091	1,484	1,460	Q30
CCP5	60,036,496	40,370	1,487	1,460	Q30
CCP6	50,551,760	34,002	1,486	1,460	Q30
CCP7	49,725,748	33,615	1,479	1,460	Q31
CCP8	44,339,720	29,627	1,496	1,483	Q31
CCP9	51,176,982	34,299	1,492	1,490	Q31

KCP, Korean chili pepper; CCP, Chinese chili pepper
